# Supplementary material for: Wnt1 silences chemokine genes in dendritic cells and induces adaptive immune resistance in lung adenocarcinoma
Source: Nat Commun. 2019 Mar 29;10:1405. doi: 10.1038/s41467-019-09370-z (PMC6441097; doi:10.1038/s41467-019-09370-z)
Supplement: Supplementary file 1 — Supplementary Information [file 41467_2019_9370_MOESM1_ESM.pdf]

Wnt1 silences chemokine genes in dendritic cells and induces adaptive immune resistance in lung adenocarcinoma

Kerdidani et al.

Supplementary Figure 1

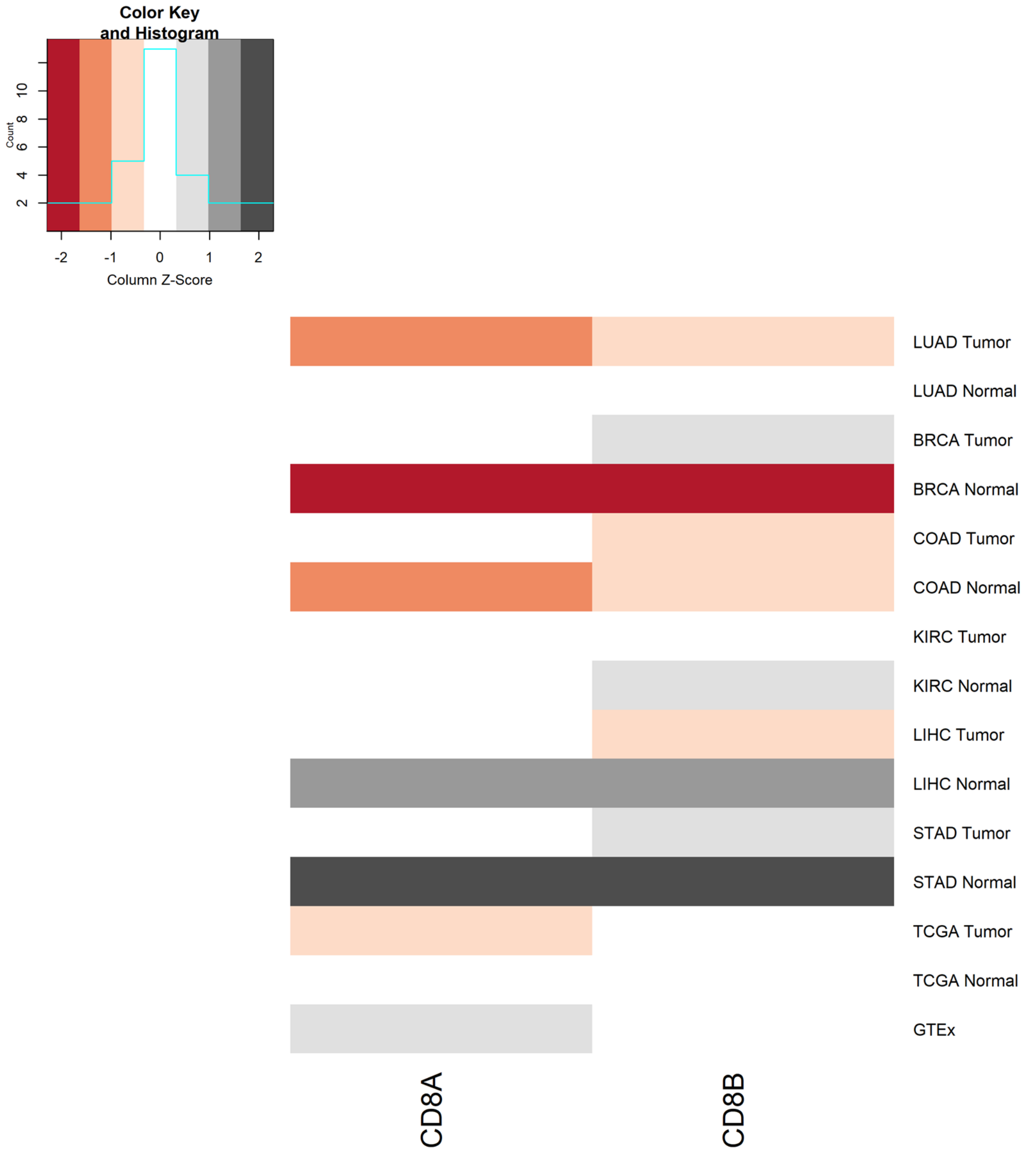

Figure S1. Heatmaps of Wnt1-CD8a, Wnt1-CD8b correlation z-scores in TCGA. Datasets from RNA sequencing analysis of paired tumor and normal tissues were analyzed for WNT1 and CD8a, CD8b. Correlation z-scores shown in heatmap. Genotype-Tissue Expression (GTEx) datasets were used as control. Column side annotations are gene names and row side annotations are cancer names. LUAD: Lung adenocarcinoma, BRCA: Breast Carcinoma, COAD: Colon Adenocarcinoma, KIRC: Kidney Renal Clear Cell Carcinoma , STAD: Stomach Adenocarcinoma , LIHC: Liver Hepatocellular Carcinoma.

Supplementary Figure 2

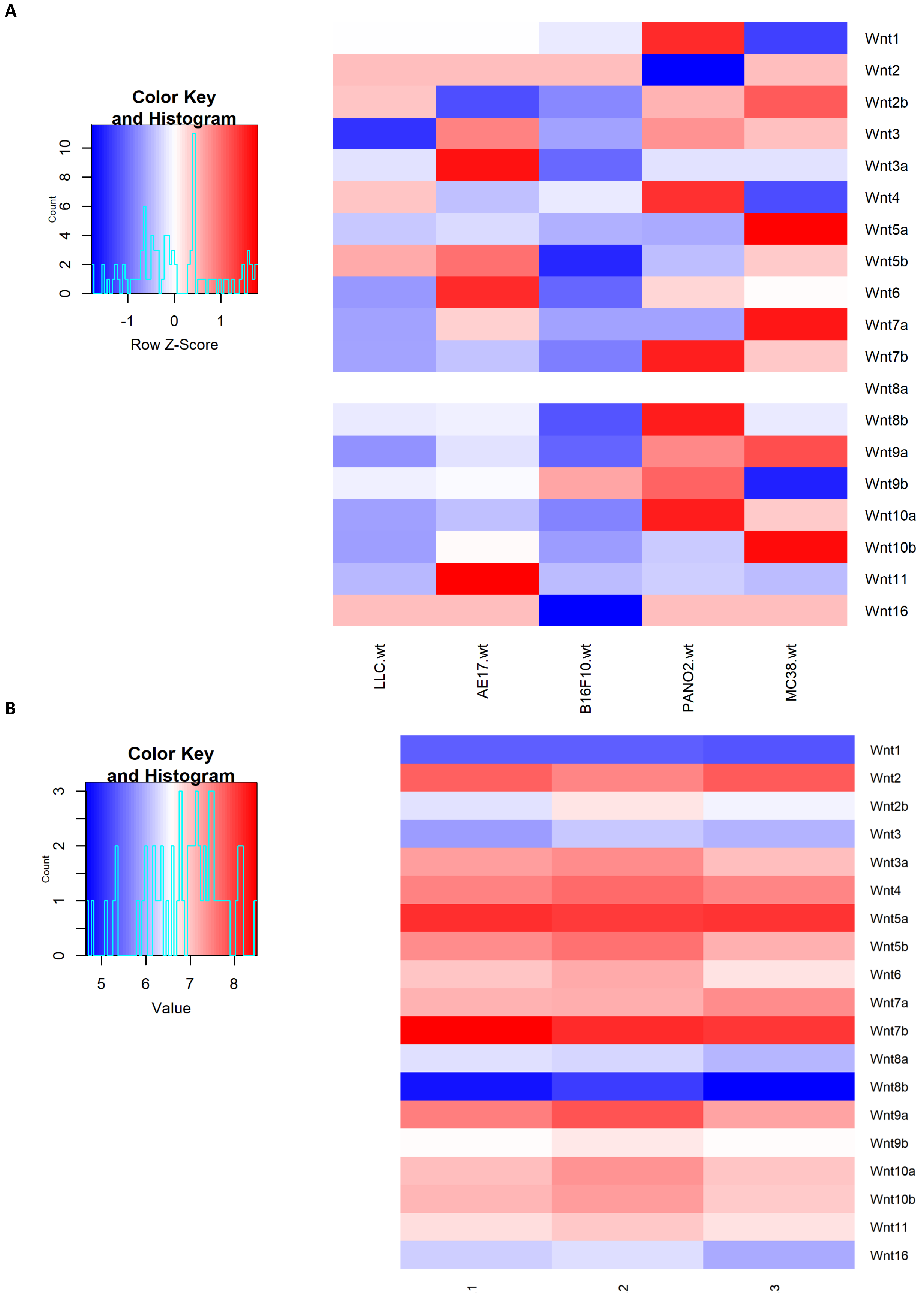

Figure S2. Microarray heatmaps of Wnt ligand expression by LLC cells in vitro and in vivo. Analysis of gene expression profiles was performed on microarray datasets from GSE. **A,** Microarray heatmap of Wnts expressed by several murine cancer cell lines, including LLC cells (GSE58188). Column side annotations are names of murine cancer cell lines and row side annotations are names of Wnt ligands. **B,** Microarray heatmap of Wnts expressed by LLC lung tumors (n=3 biological replicates) (GSE36568). Column side annotations are serial numbers of individual mice and row side annotations are names of Wnt ligands.

Supplementary Figure 3

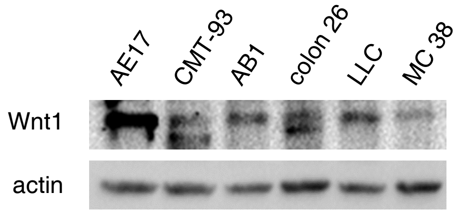

Figure S3. Comparative immunoblot analysis of Wnt1 in several murine cancer cell lines. The analysis was performed using anti-WNT1 antibody. B-actin was used as loading control. Source data are provided as a Source Data file.

Supplementary Figure 4

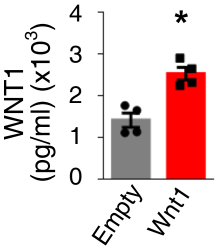

Figure S4. Extracellular Wnt1 (ELISA) in LLC lung tumors. Wnt1 overexpressing versus control (Empty) LLC lung tumors were resected, cut into small pieces and cultured for 16 hours. Wnt1 protein was quantified in culture supernatants by ELISA. Error bars represent mean with SEM. Data are representative of two independent experiments with 4 mice per group. *$p < 0.05$, with Mann Whitney U. Source data are provided as a Source Data file.

Supplementary Figure 5

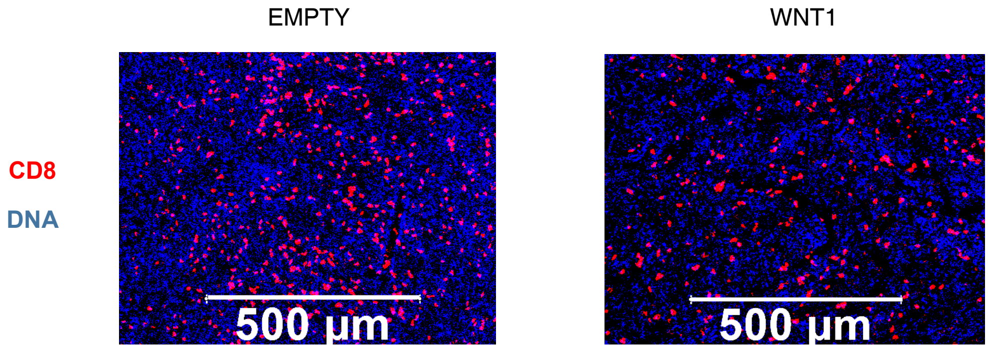

Figure S5. Confocal images of immunofluorescence staining for CD8 on lung LLC tumors. Representative images of CD8 stained lung tumor sections from Wnt1-overexpressing LLC (Wnt1) versus control (Empty) tumors.

Supplementary Figure 6

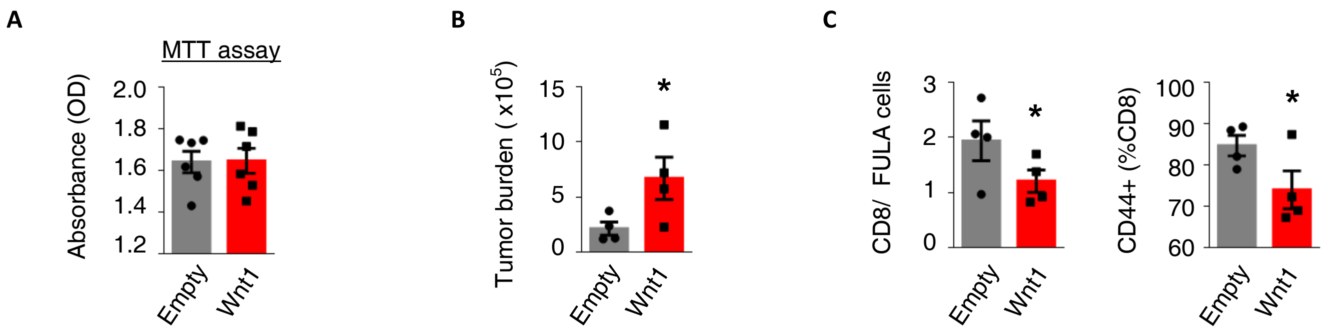

Figure S6. Growth of a Wnt1-overexpressing autochthonous lung adenocarcinoma cell line. The FULA (FVB Urethane-induced Lung Adenocarcinoma) cell line, derived from autochthonous urethane-induced lung tumor, was transduced with Wnt1 (Wnt1) or Empty (Empty) viral vectors. A, In vitro proliferation (MTT assay). B, Tumor burden (total absolute number of Fula cells) 14 days after intrapulmonary implantation in syngeneic FVB mice. C, Numbers of intratumoral $CD8^+$ T cells per cancer cell and T cell CD44 expression. Error bars represent mean with SEM. Data are representative of two independent experiments with 4-5 mice per group. *p < 0.05 with t-test. Source data are provided as a Source Data file.

Supplementary Figure 7

A

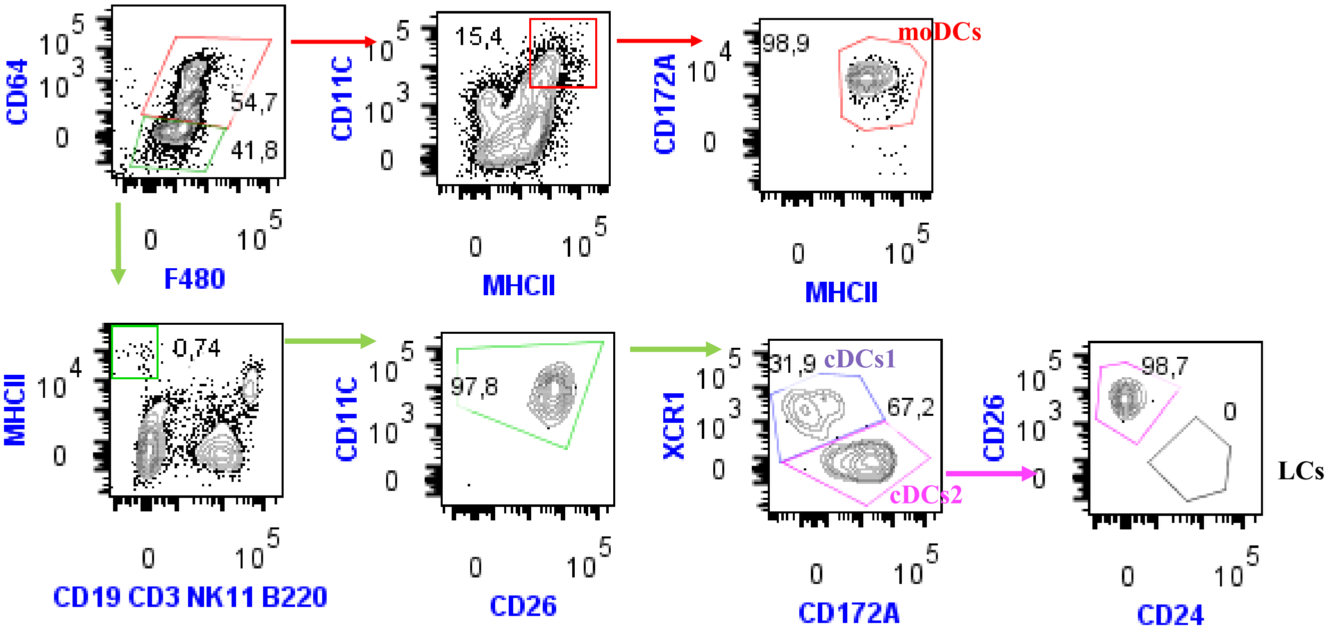

B

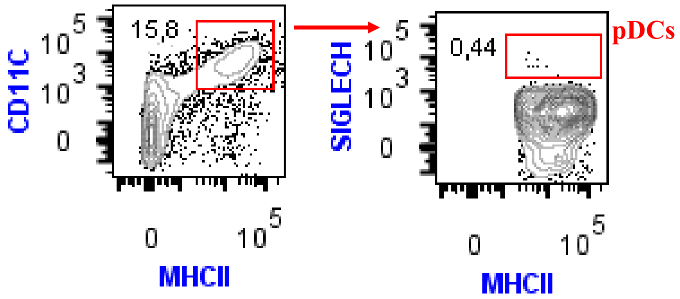

C

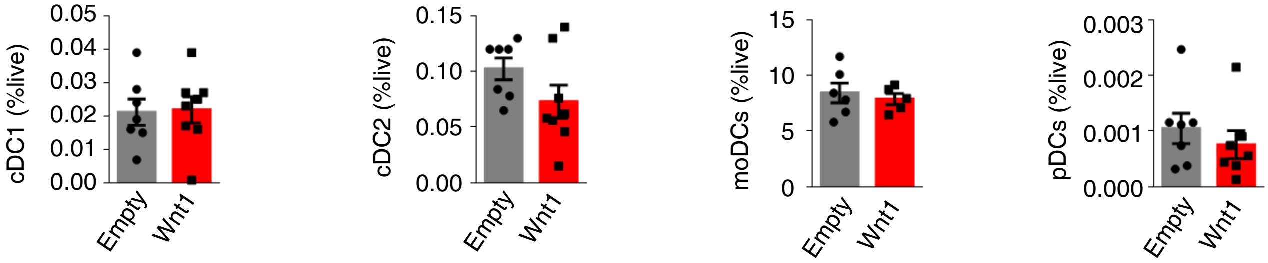

Figure S7. Gating strategy for FACS analysis of tumor infiltrating DC subsets and moDCs . WNT1-overexpressing and control (Empty) LLC cells were implanted intrapulmonary. On day 14 lung tumors were excised and dispersed. **A,** Gating of intratumoral cDCs and moDCs (Guilliams et al., 2016, Immunity 45, 1–16). cDCs1 were gated as XCR1^{+} cells, while cDCs2 as CD172a^{+} cells among CD64^{-}MHCII^{+}CD3^{-}CD19^{-}B220^{-}NK1.1^{-} CD11c^{+}CD26^{+} cells. CD26^{-}CD24^{+} Langerhans cells (LCs) were not identified in any LLC tumor. moDCs were gated as CD11c^{+}MHCII^{+}CD172^{+} cells among CD64^{+} F4/80^{+} cells. B, Gating of intratumoral pDCs. pDCs were gated as SIGLECH^{+} cells among CD11c^{+}MHCII^{+} cells. C, Percentages of DC subsets among total live cells. Error bars represent mean with SEM. Data are representative of two independent experiments with 6-8 mice per group. Source data are provided as a Source Data file.

Supplementary Figure 8

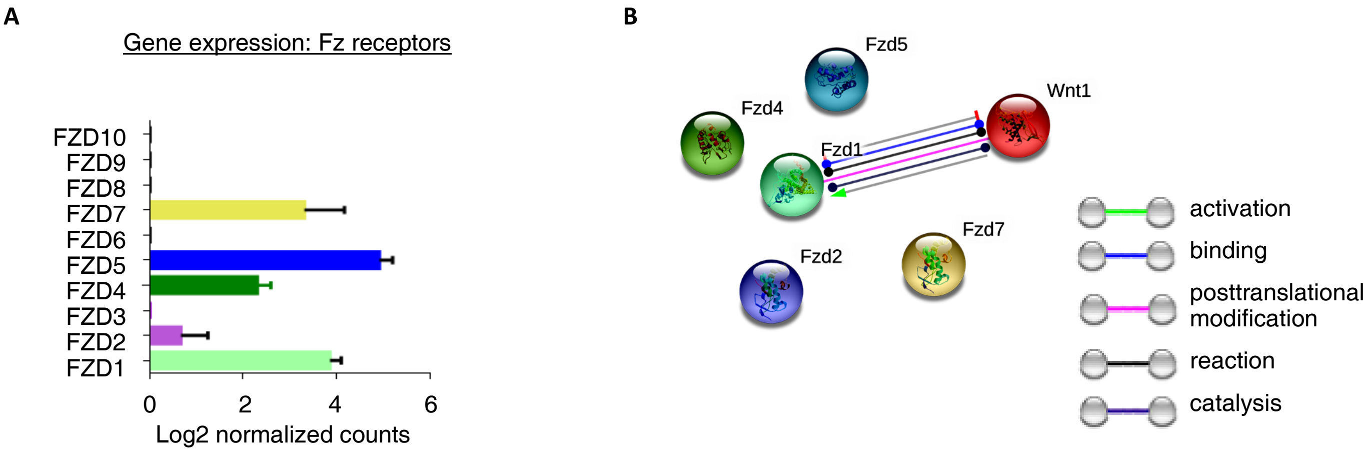

Figure S8. Frizzled receptor expression by intratumoral cDCs. A, Barplot of raw expression values (log2 read counts) of Frizzled receptors in cDCs FACS sorted from lung LLC tumors. Mean±SEM are shown. **B,** Network of Wnt1 and Frizzled receptors, inferred from String database (network indicates experiments with high confidence interaction score (0.700).

Supplementary Figure 9

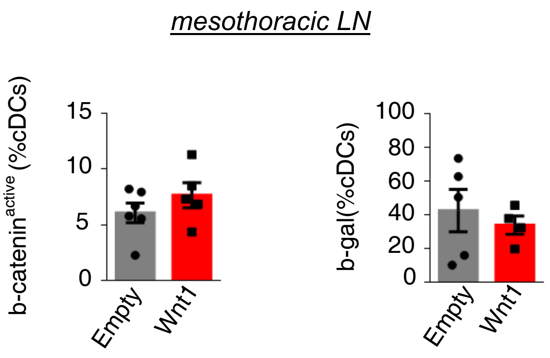

Figure S9. Wnt/b-catenin pathway activation in tumor draining lymph nodes. Wnt1-overexpressing LLC versus control (Empty) cells were implanted intrapulmonary in WNT pathway reporter mice (Axin2^{LacZ}). On day 14 mesothoracic lymph nodes were excised and dispersed. Active b-catenin and b-galactosidase expression among nodal cDCs were quantified by FACS. Mean ± SEM are shown. Data are representative of 2 independent experiments (n=4-6 mice per group). Source data are provided as a Source Data file.

Supplementary Figure 10

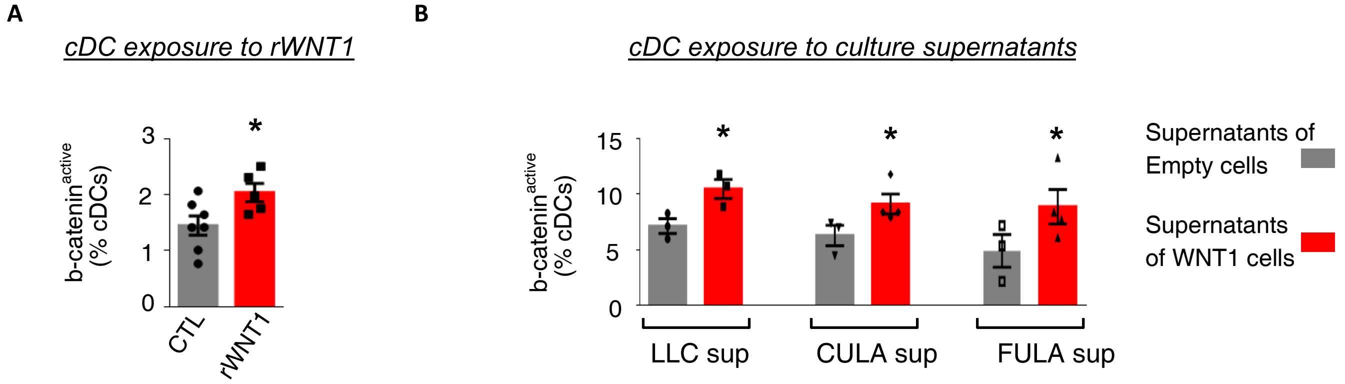

Figure S10. B-catenin activation induced by paracrine WNT1 signaling in vitro. cDCs were sorted from spleens of healthy mice and exposed to: A, 100ng/ml rWNT1 or B, culture supernatants from WNT1 overexpressing versus control (Empty) cancer cells. The following cell lines were transduced with WNT1 overexpressing or Empty viral vectors and used to obtain culture supernatants: LLC cells, C57BL/6-derived urethane-induced lung adenocarcinoma (CULA cells), and FVB-derived urethane-induced lung adenocarcinoma (FULA cells) (Agalioti et al., 2017, Nat Commun 8, 1505). Graphs depict cDCs with active b-catenin percent total cDCs assessed by FACS. Results are representative of 5 independent experiments. Biological replicates are shown. Error bars represent mean with SEM. *p < 0.05 with t-test (B). Source data are provided as a Source Data file.

Supplementary Figure 11

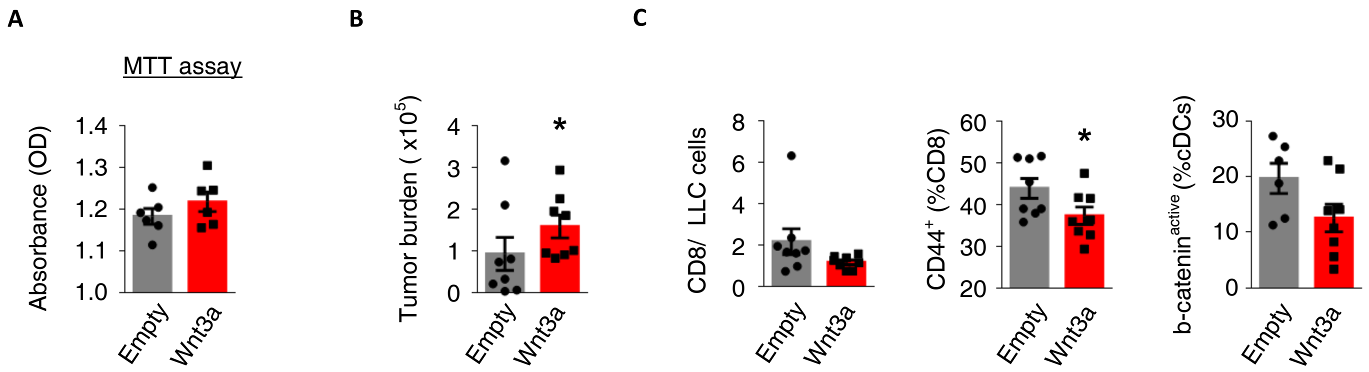

Figure S11. Growth of Wnt3a-overexpressing LLC cells. LLC cells were transduced with Wnt3a (Wnt3a) or Empty (Empty) viral vectors. A, In vitro proliferation (MTT assay). B, Tumor burden (total absolute number of LLC cells) 14 days after intrapulmonary implantation in syngeneic mice. C, Numbers of intratumoral $CD8^+$ T cells per cancer cell, T cell CD44 expression and b-catenin activation in cDCs. Error bars represent mean with SEM. Data are representative of two independent experiments with 5-8 mice per group. *$p < 0.05$ with Mann Whitney U. Source data are provided as a Source Data file.

Supplementary Figure 12

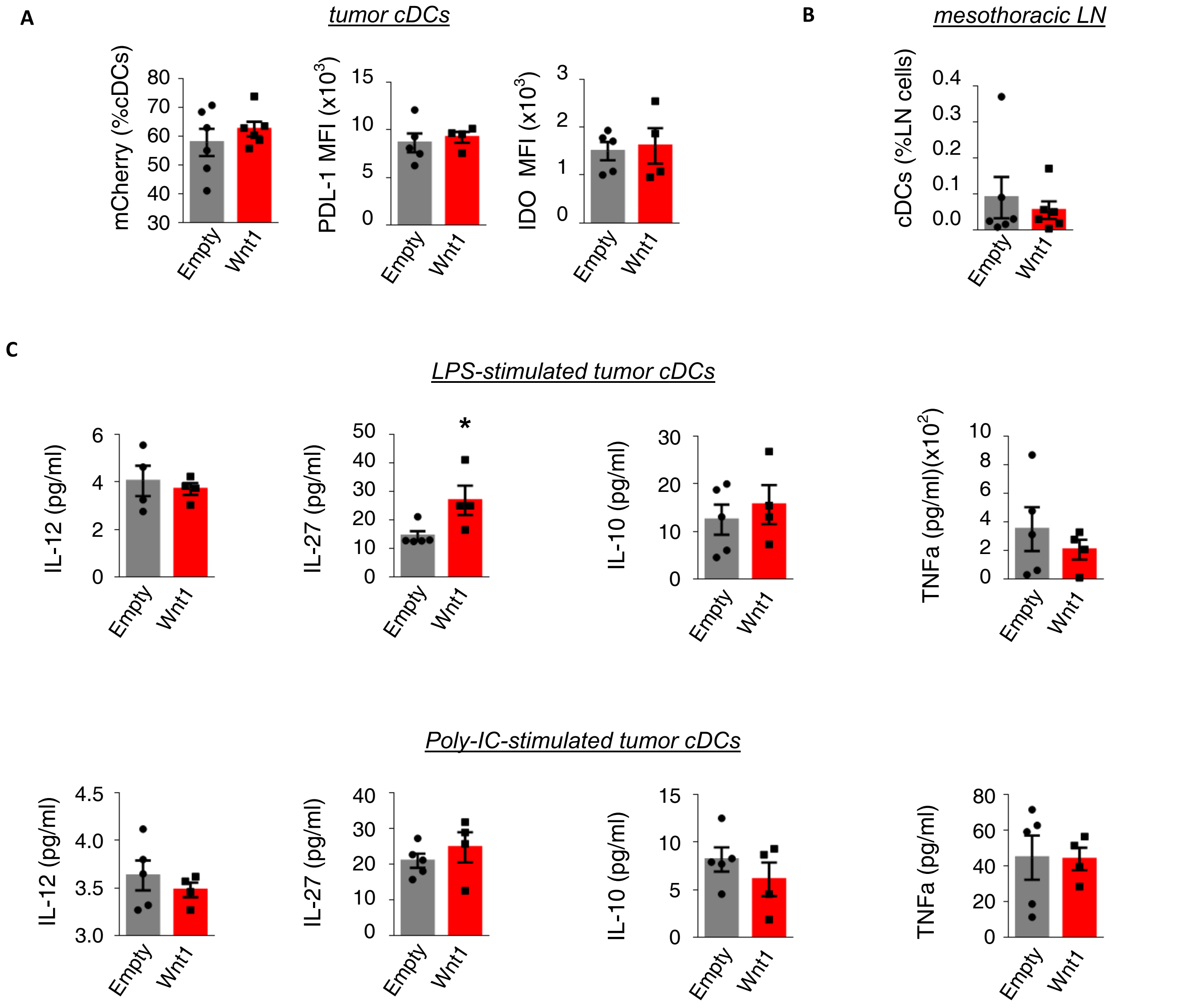

Figure S12. Phenotypic and functional characterization of intratumoral lung cDCs. Wnt1-overexpressing mCherry-LLC (Wnt1) versus control (Empty) cells were implanted intrapulmonary in syngeneic mice. On day 14 lung tumors and mesothoracic lymph nodes (LN) were obtained, mechanically dispersed and filtered. cDCs were FACS gated/sorted as $SS^{low}Lin^{-}Ly6C^{-}MHCII^{+}CD11c^{high}$. **A,** Tumor antigen capture capacity of intratumoral cDCs assessed by mCherry staining (Left). Expression of the inhibitory molecule PDL-1 (Middle) and of the immunosuppressive enzyme indoleamine (IDO) (Right). **B,** Numbers of LN cDCs. **C,** FACS sorted intratumoral cDCs were cultured for 48h in the presence of TLR ligands (LPS 1ug/ml or poly/IC 25ug/ml). Cytokine release in culture supernatants was measured by FACS using the bead-based "LEGENDplex multi-analytic assay". **A-C,** Mean±SEM are shown. **A, B,** Data are representative of two independent experiments with n=4-6 mice per group. **C,** Data are cumulative (biological replicates) of one experiment (n=5 mice per group). *p<0.05, with Mann Whitney U. Source data are provided as a Source Data file.

Supplementary Figure 13

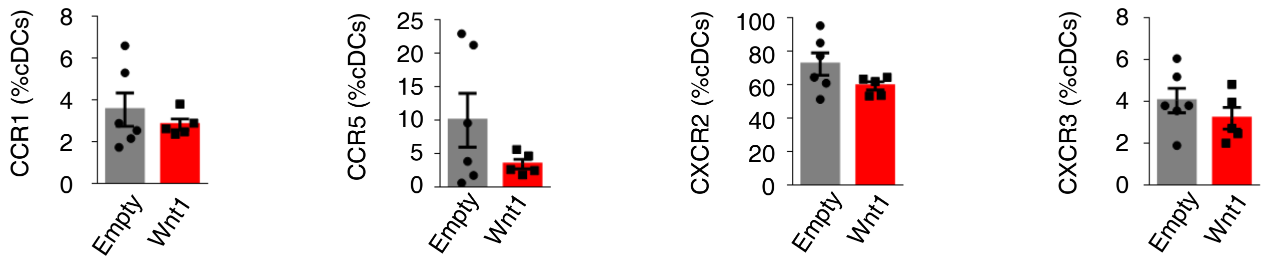

Figure S13. Chemokine receptor expression by intratumoral cDCs. Wnt1-overexpressing (Wnt1) versus control (Empty) LLC cells were implanted intrapulmonary in syngeneic mice. On day 14 lung tumors were excised and dispersed. Chemokine receptor expression was analyzed by FACS. cDCs were gated as shown in Fig. S7**.** Graphs depict chemokine receptor positive cDCs percent total cDCs. Mean±SEM are shown. Data are two independent experiments with n=4-6 mice per group. Source data are provided as a Source Data file.

Supplementary Figure 14

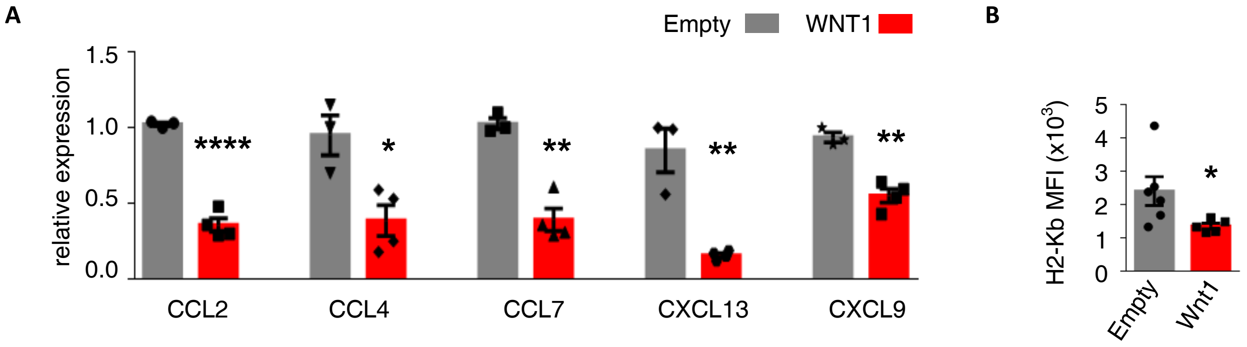

Figure S14. Validation of RNAseq dataset analysis. Reduced chemokine gene expression and H2-Kb expression in cDCs of WNT1 overexpressing (WNT1) versus control (Empty) lung LLC tumors was validated in distinct cohorts of mice. A, qPCR analysis of chemokine gene expression in sorted pooled cDCs (n=3 mice per group). Graphs depict expression relative to B2M. B, FACS analysis of H2-Kb in intratumoral cDCs. Graph depicts positive cDCs percent total cDCs. Dots indicate biological replicates. Mean±SEM are shown. Data are representative of two independent experiments . *p<0.05, **p<0.01, ****p<0.0001 with t-test. Source data are provided as a Source Data file.

Supplementary Figure 15

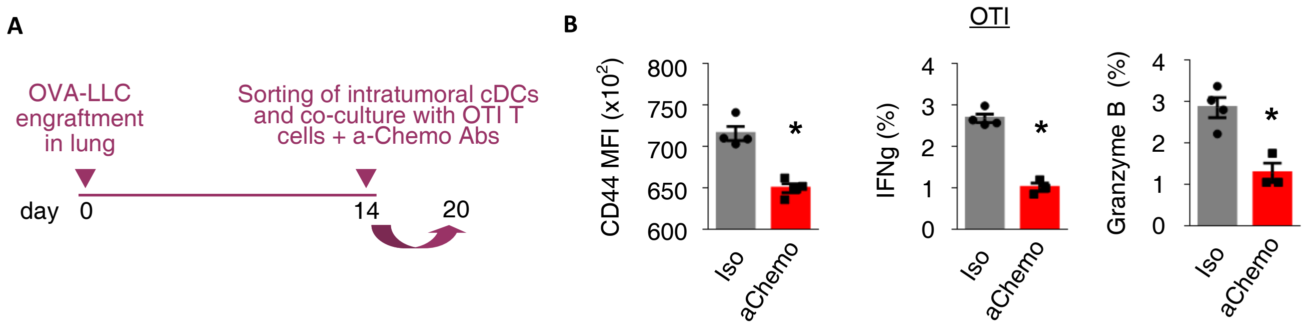

Figure S15. Chemokine blockade in vitro constrains intratumoral cDC-induced T cell priming. cDCs were FACS sorted from lung OVA-LLC tumors and co-cultured with naïve OTI T cells. A, Schematic representation of experimental design. B, T cell activation was assessed by CD44, IFNg and Granzyme B expression by FACS. Dots indicate biological replicates. Mean±SEM are shown. Data are representative of two independent experiments, n=3-4 mice per group. *$p<0.05$ with Mann Whitney U. Source data are provided as a Source Data file.

Supplementary Figure 16

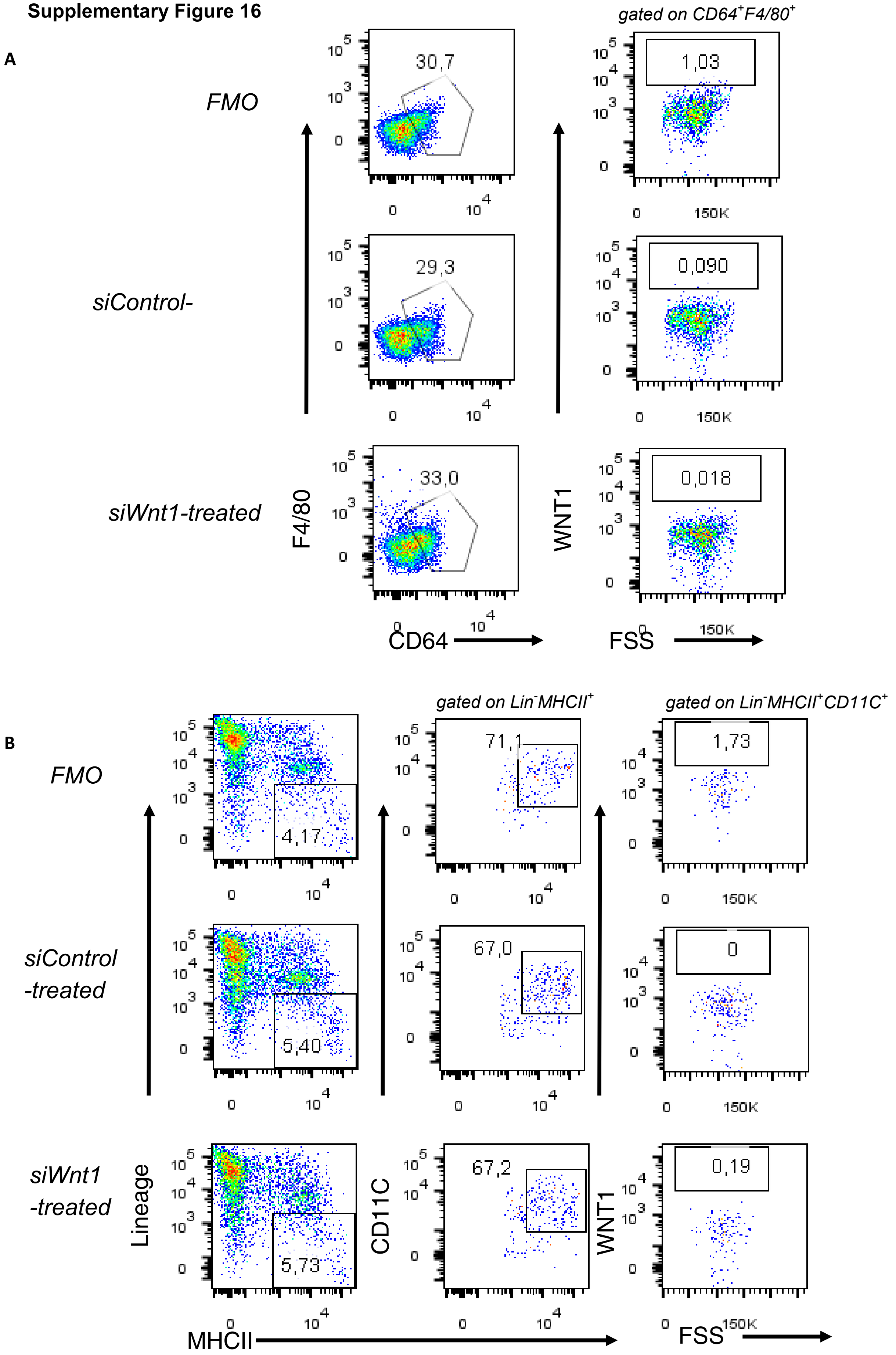

Figure S16. Wnt1 expression in intratumoral macrophages and cDCs. LLC lung tumors from siWnt1 versus siControl treated mice were lysed and Wnt1 expression was measured by intracellular staining in A, F4/80$^+$CD64$^+$ macrophages, B, CD11c$^+$MHCII$^+$ cDCs. Lineage cocktail consisted of Mabs: CD3, CD19, NK1.1, Ly6C, Ly6G, Sig-lecF, SiglecH. FMO (Fluorescent Minus One) was obtained by omitting anti-Wnt1 primary antibody, but labeling with fluorescent secondary antibody.

Supplementary Figure 17

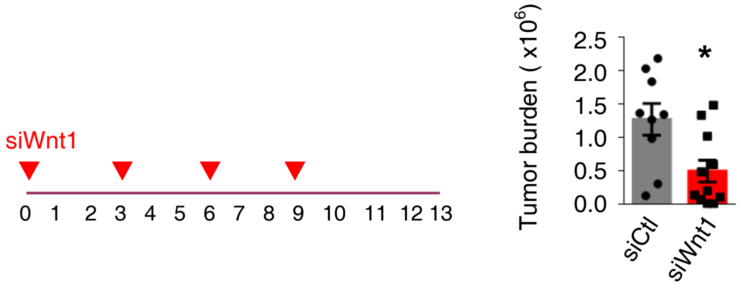

Figure S17. siWnt1 loaded nanoparticles halt growth of LLC cells in vivo. LLC cells were implanted intrapulmonary in syngeneic mice. Mice were treated with siWnt1 loaded or control nanoparticles i.p. as depicted (Left). Tumor burden (total absolute number of LLC cells) was assessed on day 13 by FACS analysis (Right). Graph depicts absolute number of tumor and mesothoracic lymph node LLC cells. Dots indicate biological replicates. Mean ± SEM are shown. Data are representative of two independent experiments, n=9-10 mice per group. *$p<0.05$ with Mann Whitney U. Source data are provided as a Source Data file.

Supplementary Figure 18

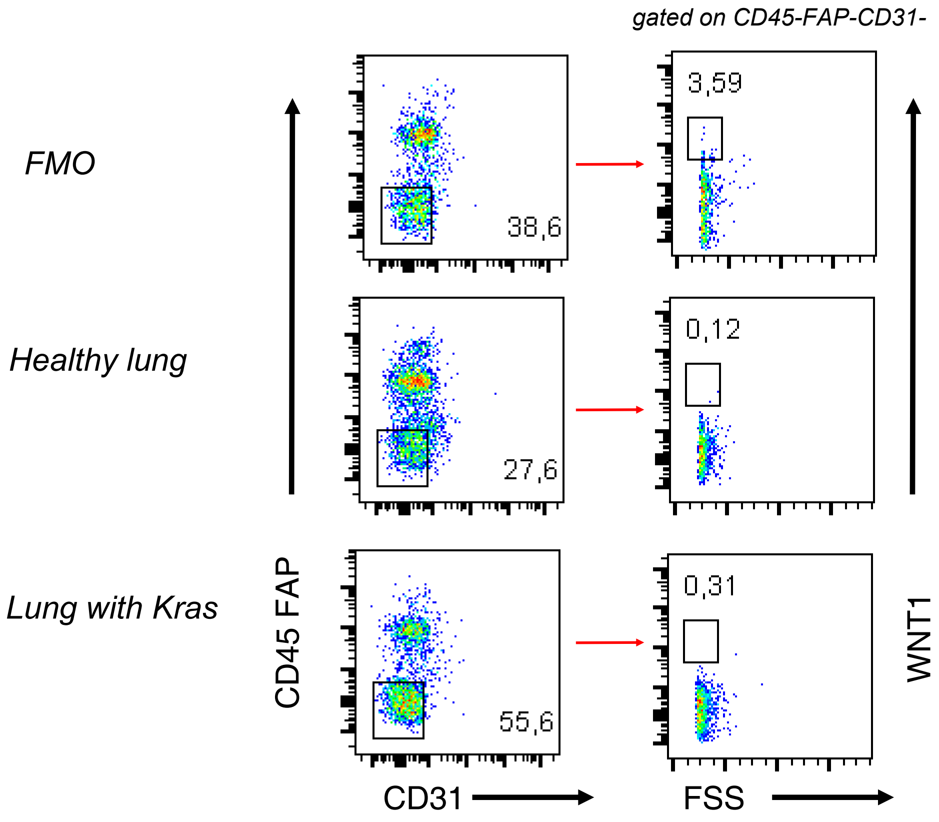

Figure S18. Wnt1 expression in KRAS driven lung tumors. Healthy and tumor bearing lungs from Kras$^{LSL-G12D}$ mice were lysed and Wnt1 expression measured by intracellular staining in live CD45$^-$FAP$^-$CD31$^-$cells. FMO (Fluorescent Minus One) was obtained by omitting anti-Wnt1 primary antibody, but labeling with fluorescent secondary antibody..

Supplementary Figure 19

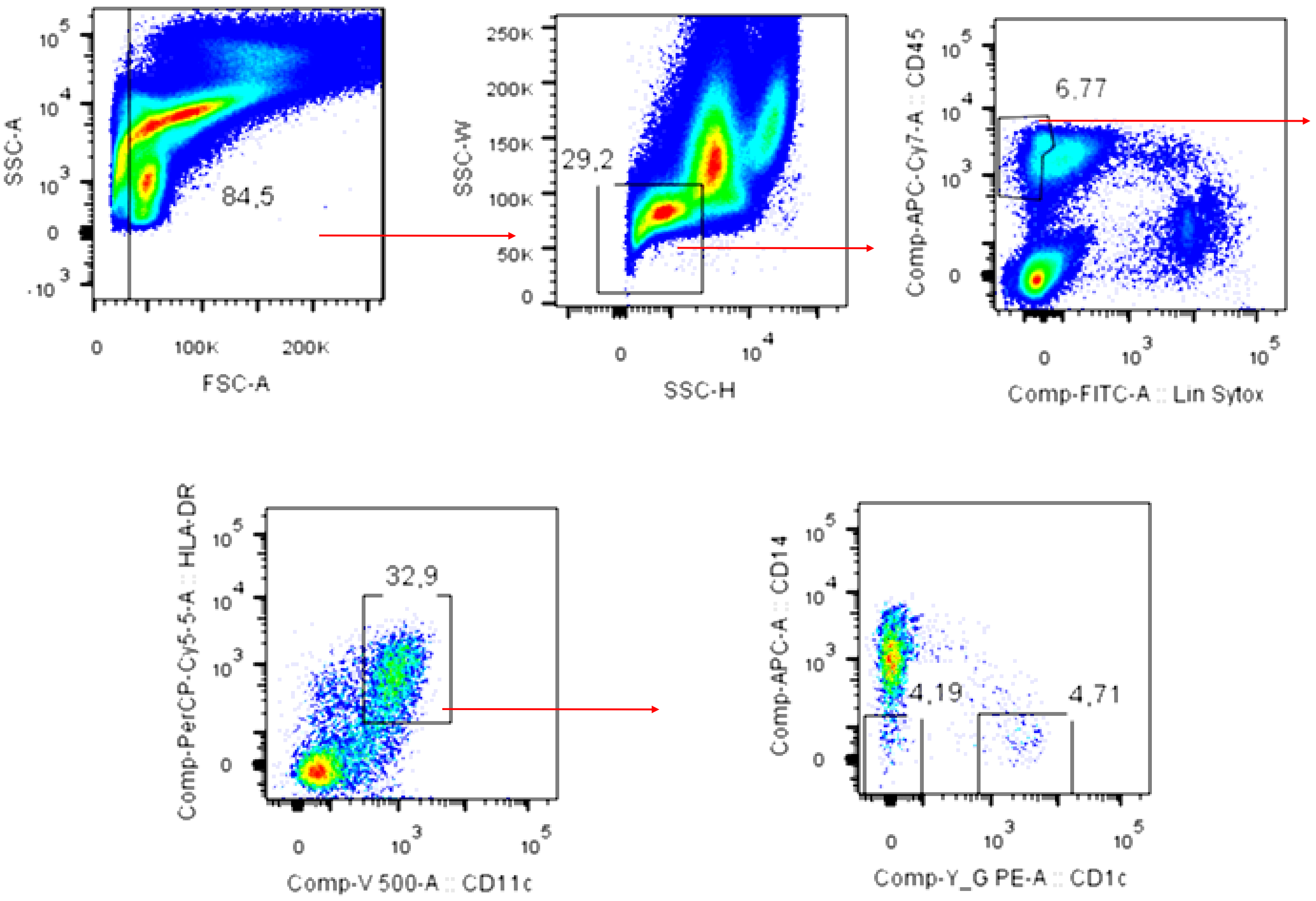

Figure S19. Gating strategy for FACS analysis and sorting of human lung cDCs. Human lung cDCs were FACS gated as CD45$^+$Lin$^-$CD14$^-$HLA-DR$^+$CD11c$^+$. Lineage cocktail consisted of Mabs against CD3 (T cells), CD19 (B cells), CD56 (NK cells). CD14 was used to exclude monocytes, macrophages and inflammatory DCs.

Supplementary Figure 20

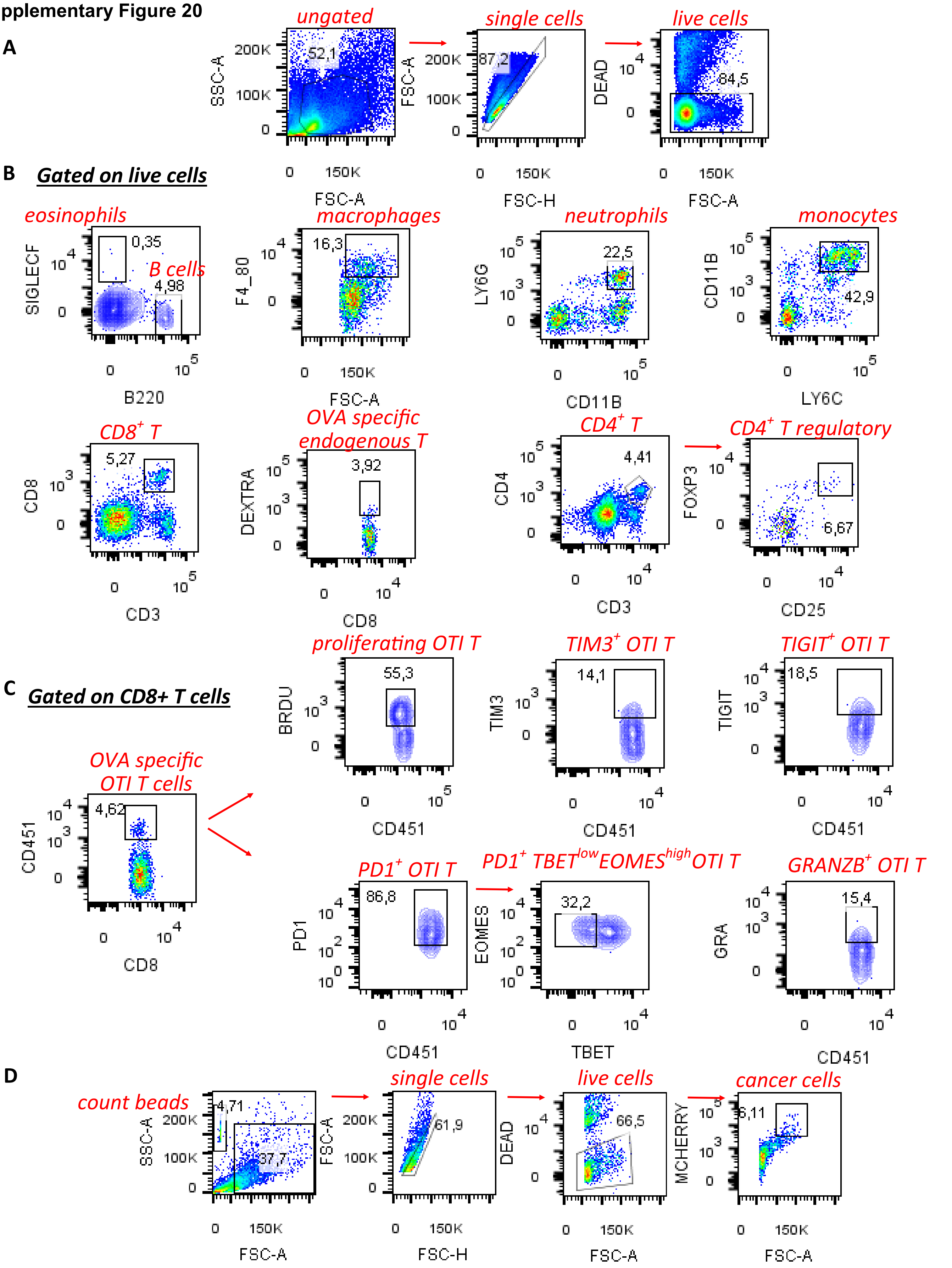

Figure S20. Gating strategies used for cell identification and characterization. (A) Gating strategy for live single cells used for all FACS analysis (Figs 2-7). (B) Gating strategy to identify eosinophils (SiglecF$^+$), B cells (B220$^+$), macrophages (FS^{high}F4/80$^+$), neutrophils (Ly6G$^+$CD11b$^+$), monocytes (CD11b$^+$Ly6C$^+$), CD8 T cells (CD8$^+$CD3$^+$), OVA specific endogenous T cells (CD8$^+$DEXTRA$^+$), CD4 T cells (CD4$^+$CD3$^+$), CD4 regulatory T cells (CD3$^+$CD4$^+$CD25$^+$Fopx3$^+$) from murine LLC lung tumors presented on Fig 2. (C) Gating strategy to analyze adoptively transferred OTI-T cells (CD8$^+$CD45.1$^+$) from murine LLC lung tumors presented on Fig 3. (D) Gating strategy to quantify tumor burden, based on enumeration of count beads and LLC cells (FS^{high}mCherry$^+$) from murine LLC lung tumors presented on Figs 2, 3, 4, 6. All gating strategies are shown for murine lung tu-

Supplementary Ta-

Information on archived FFPE tumor donors.

Patient No	Age	Smoking History	pTNM	Stage
1	61	75	T3N1M0	IIIA
2	77	50	T1aN0M0	IA
3	63	90	T2N0M0	IB
4	57	70	T2N2M0	IIIA
5	75	150	T2aN2M0	IIIA
6	64	150	T1N0M0	IA
7	63	60	T3N2M0	IIIA
8	61	80	T1aN0M0	IA
9	76	60	T3N0M0	IIB
10	70	60	T2aN0M0	IB
11	65	115	T2N0M0	IB
12	61	40	T1bN0M0	IA
13	66	60	T3N2M0	IIIA
14	61	40	T1N0M0	IA
15	70	100	T3N2M0	IIIA
16	66	60	T2N1M0	IIB
17	72	200	T2aN0M0	IB
18	41	60	T1N0M0	IA
19	61	70	T2bN1M0	IIB
20	64	60	T1N0M0	IA
21	45	75	T3N0M0	IIB
22	59	80	T2aN0M0	IB
23	69	120	T2N0M0	IB
24	69	120	T2N0M0	IB
25	69	40	T2bN1M0	IIB
26	74	75	T3N0M0	IIB
27	66	60	T2bN2 M0	IIIA
28	82	110	T3N2M0	IIIA
29	64	102	T2aN0M0	IA
30	68	80	T2bN1M0	IIB
31	73	0	T2aN1M0	IIA
32	63	113	T2N0M0	IB
33	54	90	T1N2M0	IIIA
34	71	80	T3N0M0	IIB

Table S1. Patient information. Information for consented Lung Adenocarcinoma patients who had undergone surgical resectioning as part of their treatment plan and whose paraffin -embedded tumor specimens were retrieved retrospectively and analyzed by immunohisto-chemistry and qPCR.

Supplementary Table 2

Information on fresh tumor and healthy juxtatumor donors.

Patient No	Age	Smoking History	pTNM	Stage
1	81	60	T1aN1M0	IIA
2	51	50	T2aN0M0	IB
3	52	35	T2aN2M0	IIIA
4	62	80	T2aN0M0	IB
5	59	40	T2bN1M0	IIB
6	66	60	T1bN0M0	IA
7	71	50	T2aN0M0	IB
8	73	90	T3N0M0	IIB

Table S2. Patient information. Information for consented Lung Adenocarcinoma patients who underwent surgical resectioning as part of their treatment plan and whose paired tumor/juxtatumor specimens were obtained immediately after surgery (prospectively), mechanically dispersed, cultured and/or analyzed by FACS.

Supplementary Table 3

Wnt-target Gene List
MYC, MYCN, CCND1, TCF7, LEF1, PPARD, JUN, FOSL1, PLAUR, MMP7, AXIN2, NRCAM,TCF4, CCKBR, CD44, EPHB2, BMP4, CLDN7, BIRC5, VEGFA, VEGFB, FGF18, ATOH1, MET,EDN1, MYCBP, L1CAM, ID2, JAG1, MSL1, TIAM1, NOS2, TERT, DKK1, FGF9, LBH, FGF20,LGR5, SOX9, SOX17, RUNX2, GREM1, SALL4, TNFSF11, TNFRSF11B, CYR61, SOX2, PTTG1,DLK1, FOXN1, MMP26, NANOG, POU5F1, SNAI1, FN1, FZD7, FST, WNT3A, ISL1, MMP2,MMP9, MYOD1, EN2, GJA1, GJB6, RARG, MITF, STRA6, RHOU, TNFRSF9, EFNB1, STRA6,NPP2, ISLR, TWIST1, MMP3, TBX1, TBX3, GCG, BGLAP, CDX1, PTGS2, IRX3, SIX3, SP5,NEUROD1, NKX2-2, GBX2, CACNA1G, WISP1, WISP2, IGF2, IGF1, VEGFC, ABCB1, COX2,IL6, POSTN, CDX1, CDX4, BTRC, SFRP2, PITX2, EGF, EDAR, CDH1, KRT, OVOL1, CDKN2A,CTLA4, KLF5, FGF4, CXCL8, RET, VCAN, TNFRSF19, EN2, EN1, FZD2, NLK, BIRC7, MKI67,WNT2, FZD8, CD24, EPCAM, IL27, ALDH1A1, ALDH12

Table S3. Wnt-target Gene List. The Wnt-target GeneList consists of Wnt-target genes listed on The Wnt Homepage of Stanford University (http://web.stanford.edu/group/nusselab/cgi-bin/wnt/), complemented by genes that were retrieved from the literature (J Natl Cancer Inst. 2014; 106(1), Am J Cancer Res 2015;5(3):1032-1046, Oncoimmunology. 2015 Dec; 4(12): e1052932 , Discov Med. 2015; 19 (105): 303–310).

Supplementary Table 4

Reagents and Resources.

REAGENT or RESOURCE	SOURCE	IDENTIFIER
Antibodies		
Anti-mouse CD44	BD Biosciences	553133
Anti-mouse MHCII	BD Biosciences	562928
Anti-mouse CD11b	BD Biosciences	557397
Anti-mouse CD25	BD Biosciences	553866
Anti-mouse Ly6C	BD Biosciences	553104
Anti-Ki67	Thermo Fischer Scientific	MAS-14520
Anti-Wnt1	Thermo Fischer Scientific	MA5-15544
Anti-Mouse IgG (H+L) Alexa Fluor 700	Thermo Fischer Scientific	A-21036
Anti-mouse CXCL11	Thermo Fischer Scientific	PA5-47767
Anti-mouse CXCL10	Thermo Fischer Scientific	MA5-23774
Anti-mouse CXCL9	Thermo Fischer Scientific	PA5-47020
Anti-mouse CCL3	Thermo Fischer Scientific	PA5-46951
Anti-mouse CCL4	Thermo Fischer Scientific	MA5-23742
H-2k(b) SIINFEKL	NIH	35494
Anti- mCherry	Invitrogen	M11241
Anti-mouse CD25	Biolegend	102007
Anti-mouse Foxp3	Biolegend	126407
Anti-mouse PD1	Biolegend	109103
Anti-mouse IFNγ	Biolegend	505807
Anti-mouse CD45.1	Biolegend	110723
Anti-mouse TIGIT	Biolegend	142103
Anti-mouse TIM3	Biolegend	119705
Anti- BrdU	Biolegend	339811
Anti-mouse PDL-1	Biolegend	124313
Anti-mouse CD62L	Biolegend	1004417
Anti-mouse CD3	Biolegend	100236
Anti-mouse CD19	Biolegend	115511
Anti-mouse Ly6C	Biolegend	128005
Anti-mouse Ly6G	Biolegend	127613
Anti-mouse F4/80	Biolegend	123115
Anti-mouse SiglecF	Biolegend	562680
Anti-mouse SiglecH	Biolegend	129611
Anti-mouse NK1.1	Biolegend	108719
Anti-mouse MHCII	Biolegend	107627
Anti-mouse CD11c	Biolegend	117317

Anti-mouse CD16/32	Biolegend	101302
Anti-mouse B220	Biolegend	103225
Anti-mouse CD172a (SIRPα)	Biolegend	144021
Anti-mouse CD26 (DPP-4)	Biolegend	137803
Anti-mouse CD24	Biolegend	101823
Anti-mouse/human CD45R/B220	Biolegend	103229
Anti-mouse/human CD11b	Biolegend	101245
Anti-mouse/rat XCR1	Biolegend	148220
Anti-mouse CD64	Biolegend	139311
Anti-mouse CD64	Biolegend	139315
Anti-mouse F4/80	Biolegend	123141
Anti-mouse CD195 (CCR5)	Biolegend	107015
Anti-mouse CD182 (CXCR2)	Biolegend	149315
Anti-mouse CD183 (CXCR3)	Biolegend	126531
Anti-mouse CD191 (CCR1)	Biolegend	152505
Anti-mouse H-2Kb	Biolegend	116519
Anti-rabbit IgG PE	Biolegend	406421
Anti-human CD45	Biolegend	304014
Anti-human CD8	Biolegend	301031
Anti-human CD107	Biolegend	328619
Anti-human CD3	Biolegend	317305
Anti-human CD19	Biolegend	302205
Anti-human CD56	Biolegend	318303
Anti-human CD11c	Biolegend	301633
Anti-human HLA-DR	Biolegend	307629
Anti-human CD14	Biolegend	301807
Anti-mouse F4/80	eBiosciences	12-4801-82
Anti-mouse CD3	eBiosciences	25-0031-81
Anti-mouse CD4	eBiosciences	53-0041-82
Anti-mouse Tbet	eBiosciences	12-5825-80
Anti-mouse EOMES	eBiosciences	50-4875-80
Anti-mouse phospho-S6	eBiosciences	17-9007-41
Anti-mouse phospho-AKT	eBiosciences	17-9715-41
Anti-mouse GranzymeB	eBiosciences	12-8898-80
Anti-mouse IDO	eBiosciences	46-9473-80
Anti-human CD1	eBiosciences	25-0015-41
Anti-Wnt1	Abcam	ab15251
Anti-Wnt3a	Abcam	ab19925
Anti-Actin	Santa Cruz	sc-1615
Bacterial and Virus Strains		
DH5a competent cells	In house	N/A
Retroviruses	This paper	N/A
Lentiviruses	This paper	N/A
Adenoviruses	Vector Development Lab,	N/A

Biological Samples		
Human lung cancer sample and adja-	Department of Surgery, Sotiria	N/A
Paraffin sections of human lung can-	Department of Pathology,	N/A
Chemicals, Peptides, Recombinant Proteins and Viruses		
Urethane	Sigma	CAS 51-79-6
Sytox Green Nucleic Acid Stain	Invitrogen	S7020
BD Horizon™ Fixable Viability Stain	BD Biosciences	564997
Zombie NIR™ Fixable Viability Kit	Biolegend	42315
Zombie Green™ Fixable Viability Kit	Biolegend	423111
Propidium Iodide	Sigma-Aldrich	255535-16-4
Mouse IL-2	R&D Systems	402-ML-100
Mouse Wnt-1/sFRP-1	R&D Systems	9765-WN-010
LPS e.coli	Sigma-Aldrich	011B4
Poly I/C	Amersham Biosciences	27-4732
BrdU	Thermo Fischer Scientific	B23151
mWNT3a lentiviral particles	Gen Target Inc	LVP610
Critical Commercial Assays		
Fluorescein di-β-D-galactopyranoside	Molecular Probes	F1179
Dynabeads Sheep anti-Rat	Invitrogen	11035
CD45 Microbeads mouse	Miltenyi Biotech	130-052-301
CD45 Microbeads human	Miltenyi Biotech	130-045-801
Deposited Data		
Experimental Models: Cell Lines		
LL/2 (LLC1) Lewis Lung Carcinoma	ATCC	CRL-1642
2.43 hybridoma cell line	ATCC	TIB-210
HEK lenti-X	Fousteri Lab BSRC Al. Fleming	
AE17	Kalomenidis Lab University of	
CMT-93	Kontoyiannis Lab BSRC Al.	
AB1	Kalomenidis Lab University of	
Colon 26	Kalomenidis Lab University of	

MC 38	Kalomenidis Lab University of Ath-	
platinum-E	Capetanaki Lab	
CULA	Stathopoulos Lab<br>University of Pa-	
FULA	Stathopoulos Lab<br>University of Pa-	
Experimental Models: Organisms/Strains		
OT-I TCR transgenic mice	Andreakos Lab	
B6.SJL-Ptprc^{a} mice	Jackson Laborato-	002014
B6(Cg)-*Zbtb46*$^{tm1(HBEGF)Mnz}$/J mice	Jackson Laborato-	019506
B6N.129P2-*Axin2*^{tm1Wbm}/J	Behrens Lab Uni-	
B6.129S4-*Kras*^{tm4Tyj}/J	Jackson Laborato-	008179
Oligonucleotides		
H_WNT1_1	Sigma-Aldrich	**KSPQ12012**
H_WNT10A_1	Sigma-Aldrich	**KSPQ12012**
H_WNT10B_1	Sigma-Aldrich	**KSPQ12012**
H_WNT2_1	Sigma-Aldrich	**KSPQ12012**
human U6 snRNA	Invitrogen	*Fw:*5'-CTCGCTTCGGCAGCACA-3'<br>*Rv:* 5'- AACGCTTCACGAATTTGCGT-
M_CCL2_1	Sigma-Aldrich	**KSPQ12012**
M_CCL4_1	Sigma-Aldrich	**KSPQ12012**
M_CCL7_1	Sigma-Aldrich	**KSPQ12012**
M_CCL4_1	Sigma-Aldrich	**KSPQ12012**
M_CXCL3_1	Sigma-Aldrich	**KSPQ12012**
M_CXCL9_1	Sigma-Aldrich	**KSPQ12012**
Mouse B2M	Invitrogen	*Fw:* 5'-TTCTGGTGCTTGTCTCACTGA -3' *Rv*: 5'-
siWNT1 mouse	Sigma-Aldrich	SASI_Mm01_00030630
siRNA Universal Negative Control #1	Sigma-Aldrich	
siWNT1 Human	Sigma-Aldrich	SASI_Hs01_00223473
siRNA Fluorescent Universal Negative	Sigma-Aldrich	

Recombinant DNA		
pMIGR1-OVA-IRES-eGFP	Zehn Dietmar Swiss Vaccine	
pMSCV-IRES-mCherry FP	Vignali Dario University of	
pLNC-WNT1	Jan Kitajewski Columbia Uni-	
